# Hes1 triggers epithelial-mesenchymal transition (EMT)-like cellular marker alterations and promotes invasion and metastasis of nasopharyngeal carcinoma by activating the PTEN/AKT pathway

**DOI:** 10.18632/oncotarget.5457

**Published:** 2015-10-02

**Authors:** Sheng-Chun Wang, Xiao-Lin Lin, Hui-Yan Wang, Yu-Juan Qin, Lin Chen, Jing Li, Jun-Shuang Jia, Hong-Fen Shen, Sheng Yang, Rao-Ying Xie, Fang Wei, Fei Gao, Xiao-Xiang Rong, Jie Yang, Wen-Tao Zhao, Ting-Ting Zhang, Jun-Wen Shi, Kai-Tai Yao, Wei-Ren Luo, Yan Sun, Dong Xiao

**Affiliations:** ^1^ Cancer Research Institute, Southern Medical University, Guangzhou 510515, China; ^2^ Institute of Comparative Medicine & Laboratory Animal Center, Southern Medical University, Guangzhou 510515, China; ^3^ Joint Program in Transfusion Medicine, Children's Hospital Boston, Harvard Medical School, Boston, Massachusetts 02115, USA; ^4^ Department of Pathology, Guangdong Medical University, Dongguan 523808, China; ^5^ Department of Oncology, Traditional Chinese Medicine-Integrated Hospital, Southern Medical University, Guangzhou, Guangdong 510315, China; ^6^ Department of Gastroenterology, The First Affiliated Hospital of Jinan University, Guangzhou 510630, China

**Keywords:** Hes1, nasopharyngeal carcinoma (NPC), epithelial-mesenchymal transition (EMT), cancer invasion and metastasis, PTEN

## Abstract

Overexpression of the transcriptional factor Hes1 (hairy and enhancer of split-1) has been observed in numerous cancers, but the precise roles of Hes1 in epithelial-mesenchymal transition (EMT), cancer invasion and metastasis remain unknown. Our current study firstly revealed that Hes1 upregulation in a cohort of human nasopharyngeal carcinoma (NPC) biopsies is significantly associated with the EMT, invasive and metastatic phenotypes of cancer. In the present study, we found that Hes1 overexpression triggered EMT-like cellular marker alterations of NPC cells, whereas knockdown of Hes1 through shRNA reversed the EMT-like phenotypes, as strongly supported by Hes1-mediated EMT in NPC clinical specimens described above. Gain-of-function and loss-of-function experiments demonstrated that Hes1 promoted the migration and invasion of NPC cells *in vitro*. In addition, exogenous expression of Hes1 significantly enhanced the metastatic ability of NPC cells *in vivo*. Chromatin immunoprecipitation (ChIP) assays showed that Hes1 inhibited PTEN expression in NPC cells through binding to PTEN promoter region. Increased Hes1 expression and decreased PTEN expression were also observed in a cohort of NPC biopsies. Additional studies demonstrated that Hes1-induced EMT-like molecular changes and increased motility and invasion of NPC cells were mediated by PTEN. Taken together, our results suggest, for what we believe is the first time, that Hes1 plays an important role in the invasion and metastasis of NPC through inhibiting PTEN expression to trigger EMT-like phenotypes.

## INTRODUCTION

Transcriptional factor Hes1 (hairy enhancer of split 1), a member of the transcriptional repressor family basic helix-loop-helix (bHLH), is a downstream target of the Notch signaling pathway. As a member of the bHLH family, Hes1 is involved in brain development and cell lineage decision [1–6], self-renewal and differentiation of adult and embryonic stem cells [7–13], cell survival and apoptosis [11, 14], cell differentiation [15–19], and cellular senescence [17].

Hes1 overexpression has been reported in numerous tumors including colon cancer [20–22], breast cancer [23], hepatocellular carcinoma (HCC) [24], glioma [25], non–small cell lung cancer [26], head and neck squamous cell carcinomas [27], ovarian carcinomas [28], meningiomas [29], and medulloblastomas [30], suggesting the oncogenic activity of Hes1. In addition, Hes1 upregulation has been observed throughout the intestinal tumorigenesis in APC^+/−^ mice [22], the pancreatic tumorigenesis in Kras^G12D^ mice [31], and the development of mouse papillary tumors [32]. Increasing evidence supports that Hes1 regulates cancer cell proliferation [17, 21], differentiation [17–19, 21], senescence [17, 24] and resistance to chemotherapy [33]. Our recent study also demonstrated that Hes1 was essential for the self-renewal and tumourigenicity of stem-like cancer cells in colon cancer [21].

It has been reported that Hes1 showed elevated expression in the metastatic PC-3 and PC-3M human prostate cancer cells [34]. In a preliminary study, we also observed that Hes1 expression correlated with lymph node metastasis of NPC (Supplementary Table S2). Briefly, the expression of Hes1 was higher in NPC tumors at the N2–N3 and M1 stages than tumors at the N0–N1 and M0 stage, respectively. These observations suggest that Hes1 is involved in cancer invasion and metastasis. However, the underlying mechanisms are not clear.

Invasion and metastasis are significantly aggressive phenotypes of human cancers and the most lethal attributes of cancer deaths. Prevention of tumor invasion and metastasis has attracted great attention in clinical research [35–39]. Previous studies suggest that epithelial-mesenchymal transition (EMT) is a central mechanism contributing to the invasion and metastasis of various cancers [36, 40]. Therefore, identification of key factors involved in EMT and investigation of the molecular mechanism of EMT are of critically importance for understanding tumor invasion and metastasis and developing novel interventions for metastatic cancers. In the present study, we investigate whether Hes1 is involved in EMT, and invasion and metastasis of NPC cells, as well as the underlying mechanisms. Our results showed, for the first time, that Hes1 triggered EMT-like cellular marker alterations and promoted invasion and metastasis of NPC by activating the PTEN/AKT pathway.

## RESULTS

### Hes1 overexpression was frequently detected in NPC biopsies and cell lines

**Figure 1:**
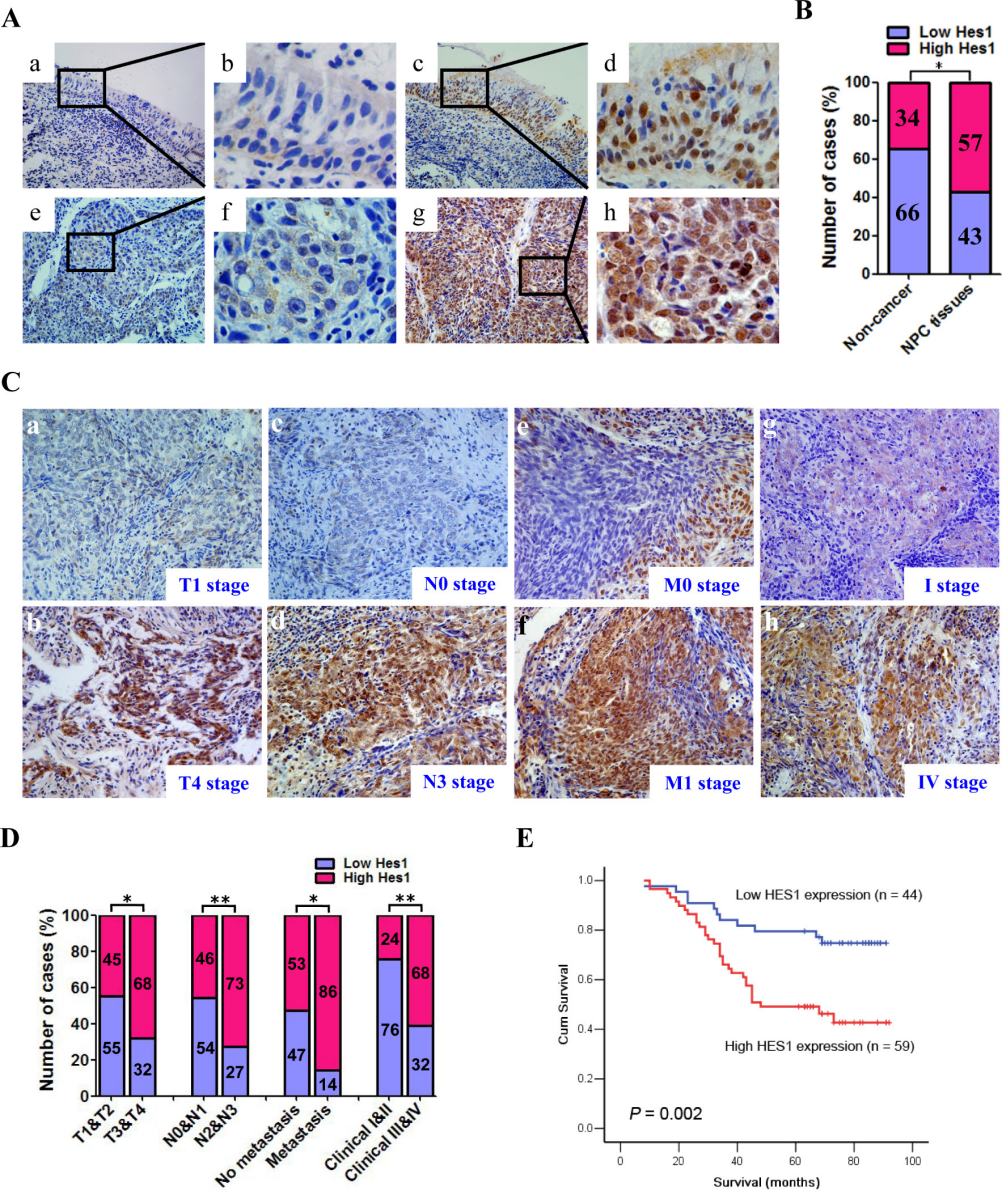
Hes1 was upregulated in nasopharyngeal carcinoma (NPC) and Hes1 upregulation was associated with the tumor progression and poor prognosis in NPC patients **A.** Hes1 expression in NPC and non-cancerous nasopharyngeal biopsies based on IHC. **a.** and **b.** Low expression of Hes1 in noncancerous nasopharyngeal biopsies; **c.** and **d.** High expression of Hes1 in noncancerous nasopharyngeal biopsies; **e.** and **f.** Low expression of Hes1 in NPC biopsies; **g.** and **h.** High expression of Hes1 in NPC biopsies. The brown staining indicates Hes1 immunoreactivity. **B.** Hes1 expression was significantly higher in the NPC biopsies than that in the noncancerous nasopharyngeal biopsies. **C.** Representative images of Hes1 expression in NPC biopsies of different TNM stages. High expression of Hes1 was observed in the T4 (b), N3 (d), M1 (f) and IV (h) stages NPC biopsies, while low expression of Hes1 was detected in the T1 (a), N0 (c), M0 (e) and I (g) stages of tumor. **D.** The numbers and percentages of high and low expression of Hes1 according to different clinicopathological features. **E.** Cumulative overall survival curves of 103 NPC patients with different Hes1 expression (high or low). The patients with high Hes1 expression had a significantly shorter overall survival than the patients with low Hes1 expression. The *P* values were calculated by log-rank test.

We first evaluated the expression of Hes1 protein in 103 paraffin-embedded, archived NPC biopsies and 29 paraffin-embedded, archived non-cancerous nasopharyngeal epithelial biopsies using immunohistochemistry (IHC) staining. High expression of Hes1 was detected in 59 of 103 NPC specimens (57.3%) and 10 of 29 non-cancerous nasopharyngeal epithelial tissues (34.5%), respectively (Figure 1A, 1B; Supplementary Table S1). Therefore, Hes1 overexpression was more frequently detected in NPC than non-cancerous nasopharyngeal epithelial biopsies.

In addition, the localization of Hes1 was found in both the nucleus and cytoplasm of NP69, CNE2, and SUNE1 cells, based on immunofluorescence assay (Supplementary Figure S1), and the distribution of Hes1 was detected in the nucleus and cytoplasm of cancer cells based on IHC (Figure 1A, 1C).

### Hes1 overexpression was associated with aggressive and/or poor prognostic phenotypes of NPC

The relationship between Hes1 expression and a number of clinicopathologic characteristics of NPC patients was summarized in Table 1. No significant association was identified between Hes1 expression and the age (*P* = 0.162), sex (*P* = 0.882), histological subtype (*P* = 0.079), and NPC recurrence (*P* = 0.718) of 103 NPC cases (Table 1). On the contrary, Hes1 expression was associated with the tumor size (T classification) (*P* = 0.018), lymph node invasion (N classification) (*P* = 0.006), metastasis (M classification) (*P* = 0.021), and clinical stage (*P* = 0.001) of 103 NPC patients (Table 1). Briefly, high expression of Hes1 was more frequently observed in T3-T4, N2-N3, M1, and III-IV tumors than T1-T2, N0-N1, M0, and I-II tumors, suggesting that Hes1 overexpression correlated with aggressive phenotypes of NPC and may be involved in the invasion and metastasis of NPC (Figure 1C, 1D and Table 1).

**Table 1:** Association between the clinicopathological features and Hes1 expression in 103 NPC patients

Variables	*n*	Hes1 expression		χ^2^	*P*
		Low (*n*, %)	High (*n*, %)		
Gender					
Female	25	11 (44.0)	14 (56.0)	0.022	0.882
Male	78	33 (42.3)	45 (57.7)		
Age (y)					
< 50	55	27 (49.1)	28 (50.9)	1.959	0.162
≥ 50	48	17 (35.4)	31 (64.6)		
Histological type					
DNKC	14	9 (64.3)	5 (35.7)	3.080	0.079
UDC	89	35 (39.3)	54 (60.7)		
T classification					
T1-T2	47	26 (55.3)	21 (44.7)	5.609	0.018
T3-T4	56	18 (32.1)	38 (67.9)		
N classification					
N0-N1	59	32 (54.2)	27 (45.8)	7.489	0.006
N2-N3	44	12 (27.3)	32 (72.7)		
M classification					
M0	89	42 (47.2)	47 (52.8)	5.353	0.021
M1	14	2 (14.3)	12 (85.7)		
Clinical stage					
I-II	25	19 (76.0)	6 (24.0)	14.944	0.000
III-IV	78	25 (32.1)	53 (67.9)		
Tumor recurrence					
No	85	37 (43.5)	48 (56.5)	0.131	0.718
Yes	18	7 (38.9)	11 (61.1)		

DNKC: differentiated nonkeratinizing carcinoma; UDC: undifferentiated carcinoma; T: tumor size; N: lymph node metastasis; M: distant metastasis

Given that Hes1 overexpression was associated with advanced NPC, we further evaluated the prognostic value of Hes1 expression for NPC patients. A negative correlation between the level of Hes1 protein expression and the overall survival of NPC patients was identified based on Kaplan–Meier analysis of the log-rank test (Figure 1E and Supplementary Table S2). The patients with higher levels of Hes1 expression had poorer overall survival than those with lower levels of Hes1 expression (*P* = 0.002; Figure 1E and Supplementary Table S2). Moreover, N/M classifications and clinical stages were also significantly associated with the overall survival of NPC patients (*P* < 0.05, all) (Supplementary Table S2). Multivariate analysis was conducted to identify independent prognostic factors for NPC patients. Multivariate Cox regression analysis showed that distant metastasis (*P* = 0.011) and tumor recurrence (*P* = 0.000) were independent prognostic factors for NPC patients, whereas Hes1 expression was not an independent prognostic factor for NPC patients (*P* = 0.053) (Supplementary Table S2). Thus, Hes1 overexpression was significantly associated with poor prognosis of NPC patients, whereas Hes1 expression was not an independent prognostic factor for NPC patients.

### Ectopic expression of Hes1 in NPC cells induced EMT-like molecular changes and enhanced cell motility and invasion

**Figure 2:**
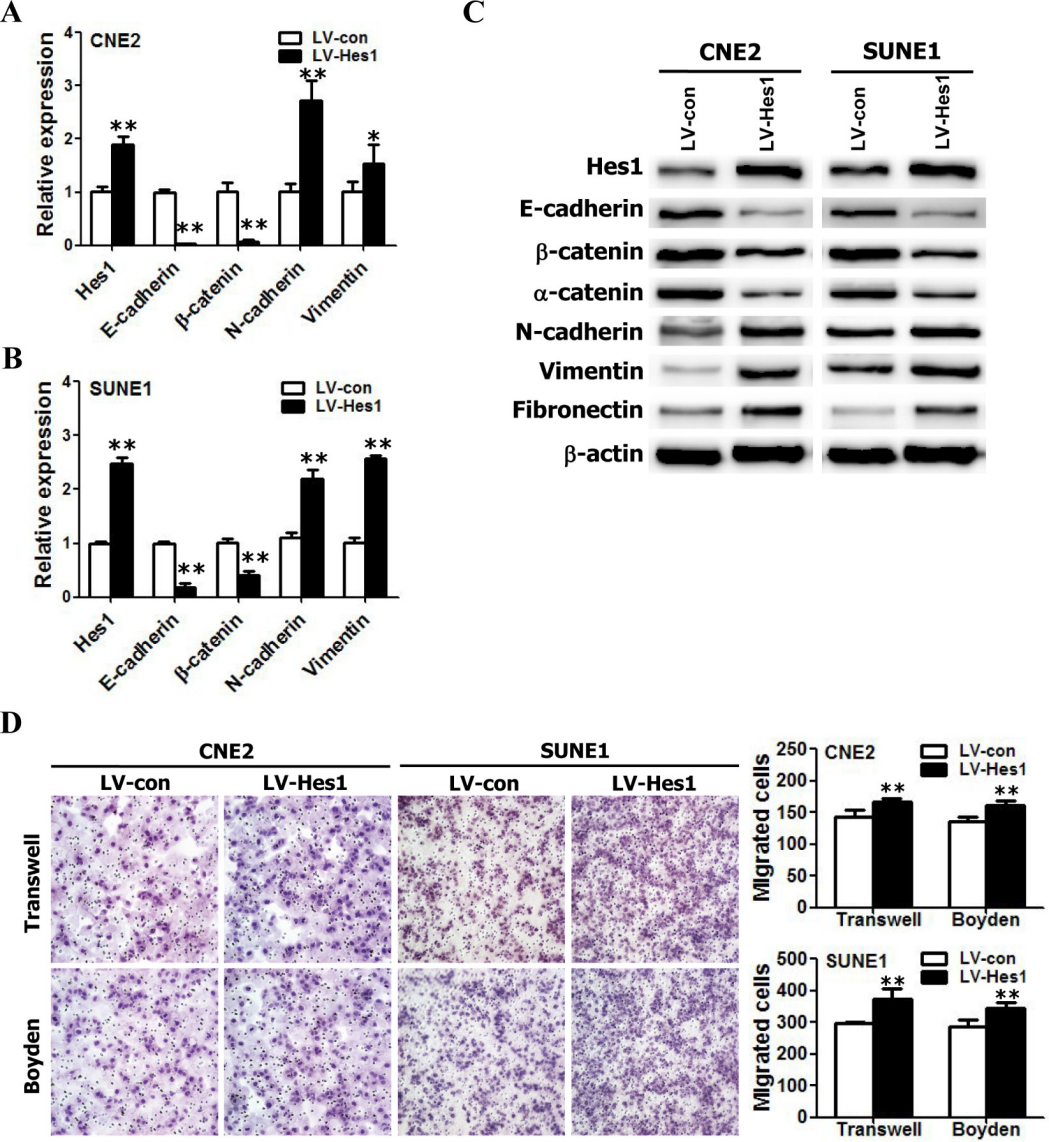
Hes1 overexpression triggered EMT-like cellular marker alterations and enhanced the migration and invasion of NPC cells *in vitro* **A–B.** The mRNA levels of E-cadherin, β-catenin, N-cadherin, and vimentin in vector- and Hes1-expressing CNE2 (A) and SUNE1 (B) cells based on qRT-PCR. **C.** The protein levels of E-cadherin, β-catenin, α-catenin, N-cadherin, vimentin, and fibronectin in vector- and Hes1-expressing CNE2 (left) and SUNE1 (right) cells based on Western blot analysis. **D.** The motile and invasive activities of Hes1-expressing CNE2 and SUNE1 cells based on transwell migration and Boyden invasion assays, respectively. Migrated cells were plotted as the average number of cells per field of view from 3 independent experiments, as described in the Methods. (Original magnification: × 200).

As mentioned above, the data from clinical NPC specimens showed that Hes1 overexpression correlated with the invasive and metastatic properties of NPC. EMT is a central mechanism contributing to invasion and metastasis of various cancers [36, 40]. To understand whether Hes1 overexpression directly induces EMT and invasion and motility of NPC cells, we examined the surface markers and phenotypic changes of NPC cells with ectopic expression of Hes1. The Hes1 transgene was successfully over-expressed in CNE2 and SUNE1 cells (Figure 2A, 2B, 2C). The qRT-PCR results demonstrated ectopic expression of Hes1 significantly reduced the expression of epithelial markers (i.e., E-cadherin and β-catenin) and significantly increased the expression of mesenchymal markers (vimentin and N-cadherin) in CNE2 and SUNE1 cells (Figure 2A, 2B). In addition, Western blotting results also revealed that Hes1-expressing CNE2 and SUNE1 cells exhibited typical EMT-like phenotypes, including downregulation of epithelial markers E-cadherin, α-catenin, and β-catenin, and upregulation of mesenchymal markers vimentin, fibronectin, and N-cadherin (Figure 2C).

EMT is one of the most important steps in tumor metastasis and advance by improving the motility and invasion of tumor cells [36, 40]. Therefore, we also examined the effects of Hes1 on the motility and invasion of NPC cells based on transwell migration and Boyden invasion assays. As shown in Figure 2D, Hes1-expressing CNE2 and SUNE1 cells displayed significantly enhanced mobility and invasion abilities in comparison with vector-expressing cells. Taken together, these results suggest that Hes1 overexpression enhanced the mobility and invasion of NPC cells *in vitro* by inducing EMT-like cellular marker alterations.

### Silence of endogenous Hes1 reversed EMT-like phenotypes and reduced the migration and invasion abilities of NPC cells

**Figure 3:**
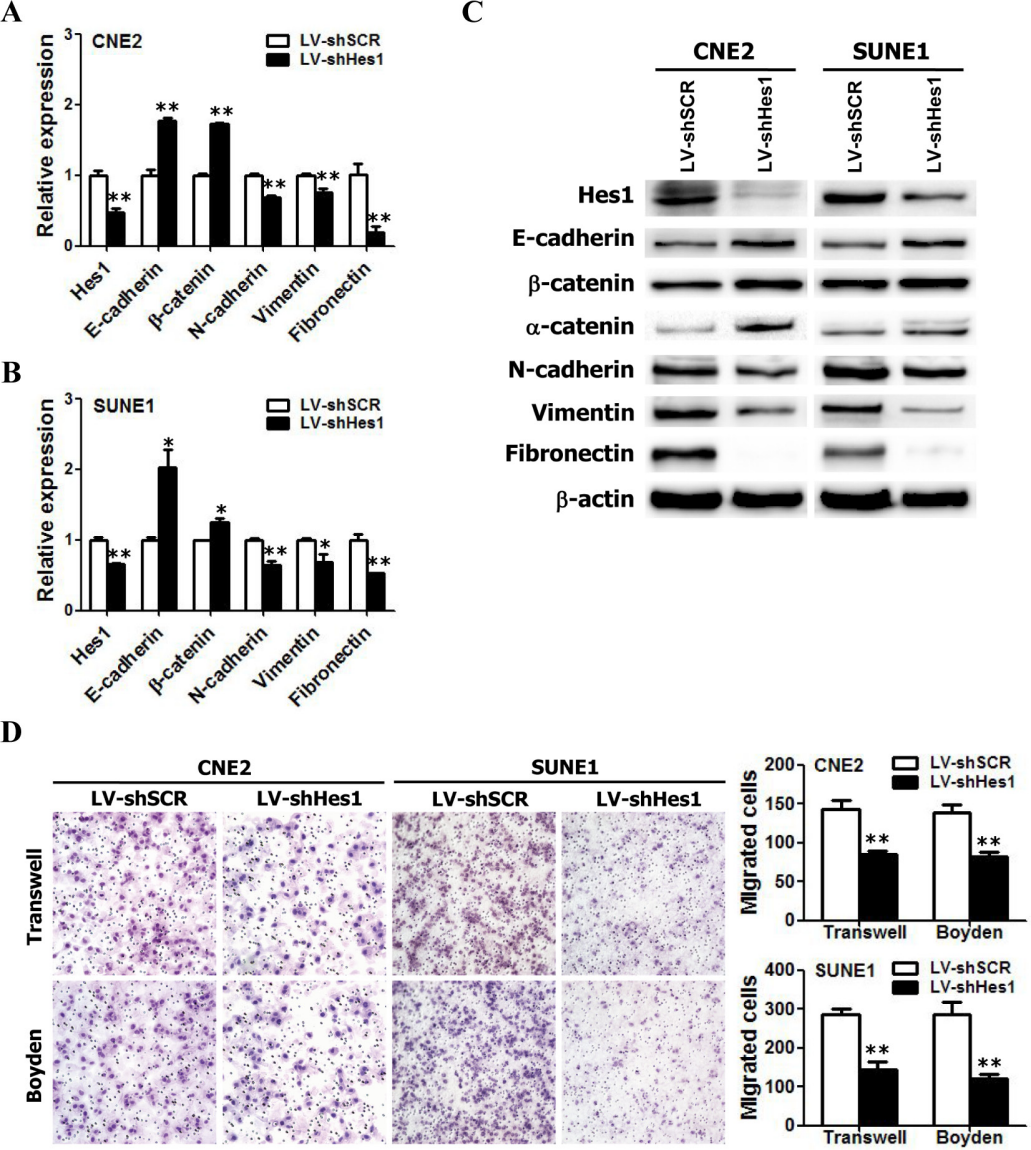
RNAi-mediated silencing of endogenous Hes1 reversed EMT-like phenotypes and inhibited the migration and invasion of NPC cells *in vitro* **A–B.** The relative mRNA levels of Hes1, E-cadherin, β-catenin, N-cadherin, and Vimentin in shHes1-expressing CNE2 and SUNE1 cells based on qRT-PCR assay. **C.** The protein levels of Hes1, E-cadherin, β-catenin, N-cadherin, and Vimentin in shHes1-expressing CNE2 and SUNE1 cells based on Western blot analysis. **D.** The motile and invasive activities of Hes1 shRNA- or scrambled shRNA–expressing NPC cells (LV-shHes1 or LV-shSCR) based on transwell migration and Boyden invasion assays, respectively. SCR: scrambled control shRNA. Original magnification: × 200.

To further examine the effects of Hes1 on EMT, migration, and invasion of NPC cells, endogenous Hes1 in CNE2 and SUNE1 cells was silenced using specific shRNA and the phenotypes were compared with wild-type NPC cells. The shRNA-Hes1 specifically knocked down endogenous Hes1 mRNA (Figure 3A, 3B) and protein (Figure 3C) expression in both CNE2 and SUNE1 cells. As indicated in Figure 3A, 3B, 3C, silencing endogenous Hes1 in CNE2 and SUNE1 cells increased the expression of epithelial markers (i.e., E-cadherin, α-catenin, and β-catenin) and concomitantly reduced the expression of mesenchymal markers (i.e., vimentin, fibronectin, and N-cadherin) at both mRNA and protein levels. Transwell migration and Boyden invasion assays also demonstrated that knockdown of endogenous Hes1 by shRNA markedly inhibited the migration and invasion of CNE2 and SUNE1 cells (Figure 3D). Taken together, suppression of endogenous Hes1 expression in NPC cells reversed EMT-like molecular changes and reduced the migration and invasion of NPC cells.

### Hes1 positively modulates the metastasis of NPC cells *in vivo*

**Figure 4:**
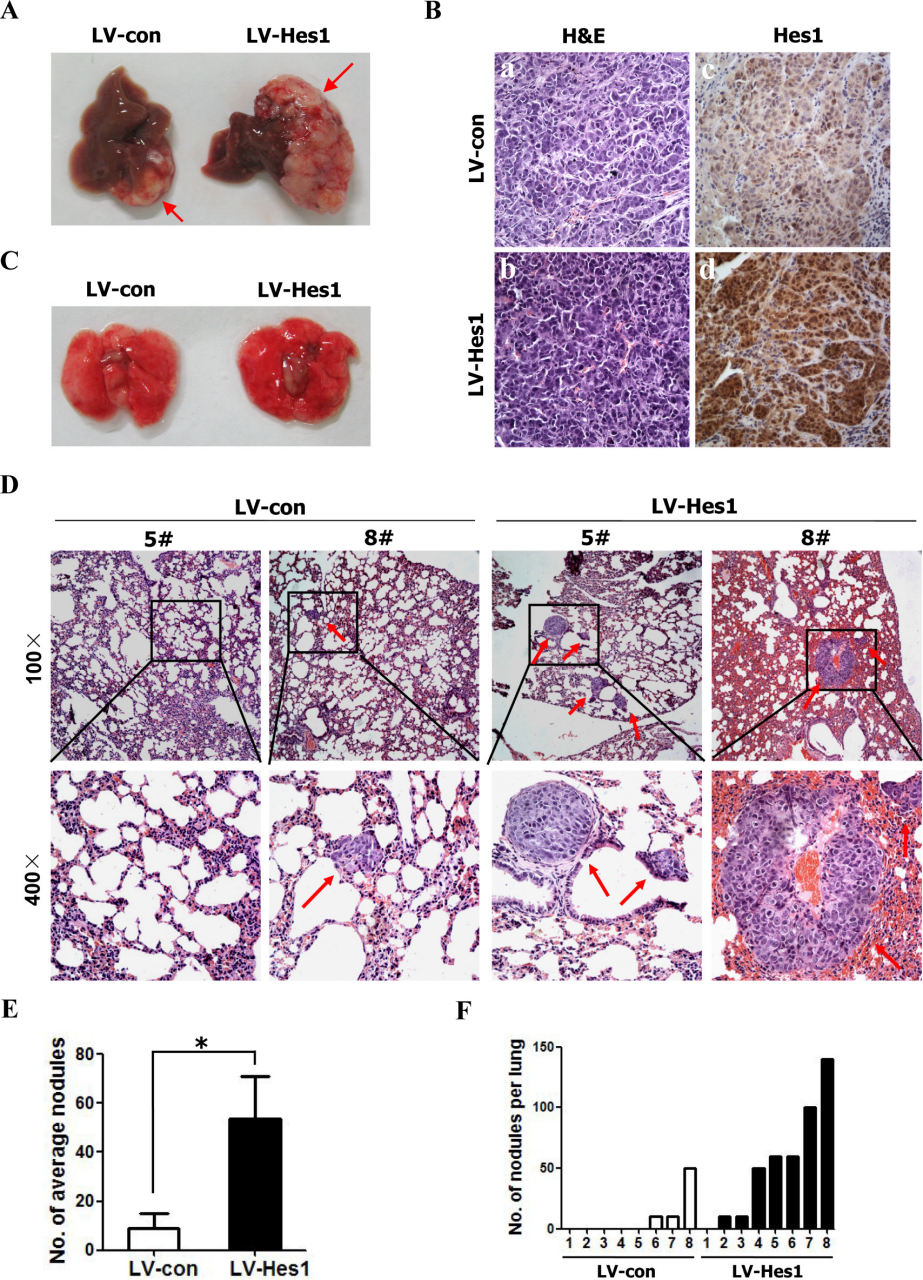
Enforced expression of Hes1 in CNE2 cells promoted metastasis **A.** Representative livers from nude mice 30 days after subcapsular liver transplantation of vector-expressing (LV-con) or Hes1-expressing (LV-Hes1) CNE2 cells (8 mice/group). Arrows indicate primarily formed tumors. **B.** H&E staining (left) and IHC analysis of Hes1 expression (right) of primary tumors in the liver of nude mice (Original magnification: × 400). **C–D.** Representative lungs (C) and H&E staining of lung sections (D) of nude mice 30 days after subcapsular liver transplantation of vector-expressing (LV-con) or Hes1-expressing (LV-Hes1) CNE2 cells. Red arrow indicates the metastatic tumor nodes. **E–F.** The number of spontaneous lung metastatic nodules in nude mice based on 10 serial sections per sample.

Local invasion and distant metastasis are common clinical features of NPC. To determine whether Hes1 improves the migration and invasion of NPC cells *in vivo*, the vector- or Hes1-expressing CNE2 cells were subcapsularly transplanted into the liver of nude mice and the formed tumors in liver and potentially metastatic tumors on lung surface were evaluated. We found that the size of tumors on liver surface derived from Hes1-expressing CNE2 cells was significantly larger than that derived from vector-expressing CNE2 cells (Figure 4A). No intrahepatic metastasis of tumors was detected in mice inoculated with vector- or Hes1-expressing CNE2 cells (data not shown). Moreover, IHC analysis revealed that Hes1 expression of primary tumors derived from Hes1-expressing CNE2 cells was significantly higher than that of primary tumors derived from vecto-expressing CNE2 cell (Figure 4B).

While metastatic tumors on lung surface derived from vector- or Hes1-expressing CNE2 cells were not detected under a dissection microscope (Figure 4C), histological examination identified an average of 23.3 ± 23.1 metastatic tumor nodules on lung surface in three of eight mice received CNE2-vector transplant (Figure 4D, 4E, 4F). In contrast, an average of 61.4 ± 46.7 metastatic tumor nodules were detected on lung surface in seven of eight mice inoculated with Hes1-expressing cells (Figure 4D, 4E, 4F) based on histological examination. Furthermore, H&E staining showed that the size of micrometastatic lesions was markedly larger in the lungs of mice injected with Hes1-expressing cells than in the mice inoculated with CNE2-vector (Figure 4D). In addition, RNAi-mediated silencing of endogenous Hes1 in CNE2 cells significantly inhibited the metastatic ability of NPC cells *in vivo* (Supplementary Figure S2). Thus, Hes1 positively regulates the metastasis of NPC cells *in vivo*.

### Hes1 inhibited the expression of PTEN by binding to its promoter region

**Figure 5:**
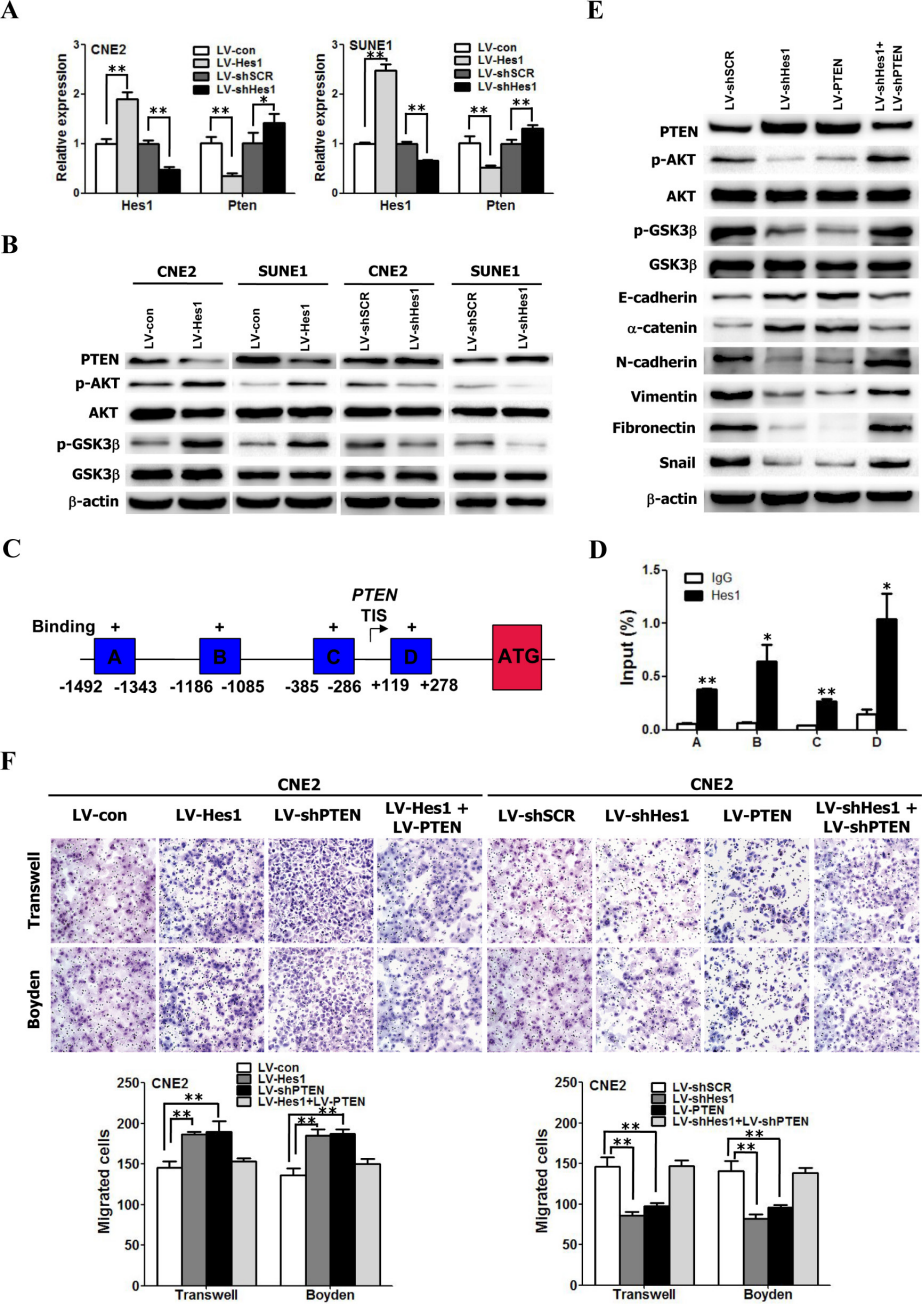
Hes1 induced EMT-like molecular changes and promoted cell motility and invasiveness through inhibiting PTEN expression **A.** The mRNA levels of Hes1 and PTEN in NPC cells transfected with different plasmids based on qRT-PCR assay. **B.** The protein levels of Hes1 and PTEN in NPC cells transfected with different plasmids based on Western blot analysis. **C.** Schematic diagram of the PTEN promoter region showing potential binding sites of HES1. Immunoprecipitated DNA was amplified by PCR using primers specific for regions A–D. The arrow indicates the transcriptional start site. TIS: transcription initiation site; ATG: the translation start codon. **D.** ChIP assay was performed using anti–Hes1 antibody or IgG antibody to identify Hes1 binding sites on the PTEN promoter in CNE2 cells. **E.** Cell extracts from CNE2 cells transfected with different plasmids were analyzed by immunoblotting with antibodies against the indicated proteins. **F.** Hes1 promoted the motility and invasion of CNE2 cells through inhibiting PTEN expression. The motile and invasive activities of the indicated CNE2 cells were analyzed using transwell and Boyden assays, respectively. Original magnification: 200 ×.

As a transcriptional repressor [17, 41, 42], Hes1 is not able to directly activate the PI3K/Akt pathway. It was speculated that Hes1–dependent activation of the PI3K/Akt pathway was mediated by transcriptional repression of an AKT-negative regulator through Hes1. It is well known that the tumor suppressor PTEN negatively regulates the PI3K/Akt pathway [43–45]. Therefore, we compared PTEN expression among Hes1-expressing, shHes1-expressing, and relative control cells. The qRT-PCR and Western blotting results revealed that Hes1 overexpression led to downregulation of PTEN mRNA and protein levels, while Hes1 silence resulted in a significant upregulation of PTEN at both mRNA and protein levels (Figure 5A, 5B), suggesting that Hes1 negatively regulates PTEN expression. We next performed chromatin immunoprecipitation (ChIP) assay to examine whether Hes1 is associated with the PTEN locus. ChIP analysis showed that Hes1 bound to the regulatory sequences in the PTEN promoter region in CNE2 cells (Figure 5C), which was validated by quantitative ChIP assays (Figure 5D). The promoter luciferase reporter assays revealed that the DNA sequence has promoter activity and Hes-1 directly regulates the promoter activity of PTEN [46]. Taken together, these results suggest that Hes1 transcriptionally inhibited the expression of PTEN by binding to its promoter region.

### Hes1 induced EMT-like molecular changes and enhanced migration and invasion of NPC cells through inhibiting the expression of PTEN

To analyze the functional correlation between PTEN and Hes1, we further examine whether stable shRNA-mediated knockdown of PTEN expression in Hes1–silenced CNE2 cells could reactivate PI3K/AKT/GSK-3β signaling pathway. As shown in Figure 5E, PTEN overexpression markedly reduced AKT and GSK-3β phosphorylation in CNE2 cells, similar as the results caused by Hes1 silencing. In addition, silencing endogenous PTEN with PTEN-shRNA in Hes1–silenced CNE2 cells increased the levels of phosphorylated AKT (Ser473) and GSK-3β, and snail (Figure 5E).

To understand whether Hes1-induced EMT and enhanced motility and invasion of NPC are mediated by PTEN, we performed gain-of-function and loss-of-function experiments. First, we examined the role of PTEN in EMT, and migration and invasion of NPC cells by re-expression of PTEN. As shown in Figure 5, gain of PTEN function through exogenous expression of PTEN increased the expression of epithelial markers (E-cadherin and α-catenin) (Figure 5E), reduced the expression of mesenchymal markers (vimentin, fibronectin, N-cadherin and snail) (Figure 5E), and inhibited the motility and invasion of CNE2 cells, which were similar as the results caused by Hes1 silencing (Figure 5F).

Subsequently, we investigated whether ectopic expression of shPTEN reverses shHes1-induced mesenchymal-epithelial transition (MET) and rescues shHes1-mediated inhibition of migration and invasion of CNE2 cells. We found that shPTEN reversed MET-like phenotypes (Figure 5E) and abrogated the inhibition of motility and invasion of CNE2 cells (Figure 5F) induced by shHes1. Furthermore, shRNA-mediated PTEN knockdown enhanced migration and invasion of CNE2 cells, similar to the results of ectopic expression of HES1 in NPC cells (Figure 5F). PTEN overexpression in Hes1-expressing cells abrogated the increased motility and invasion of CNE2 cells (Figure 5F) induced by enforced expression of Hes1. Taken together, these results suggest that PTEN is involved in Hes1-induced EMT-like phenotypes, motility, and invasion of NPC cells.

### Association among Hes1 and PTEN expression and EMT phenotypes in NPC patients

**Figure 6:**
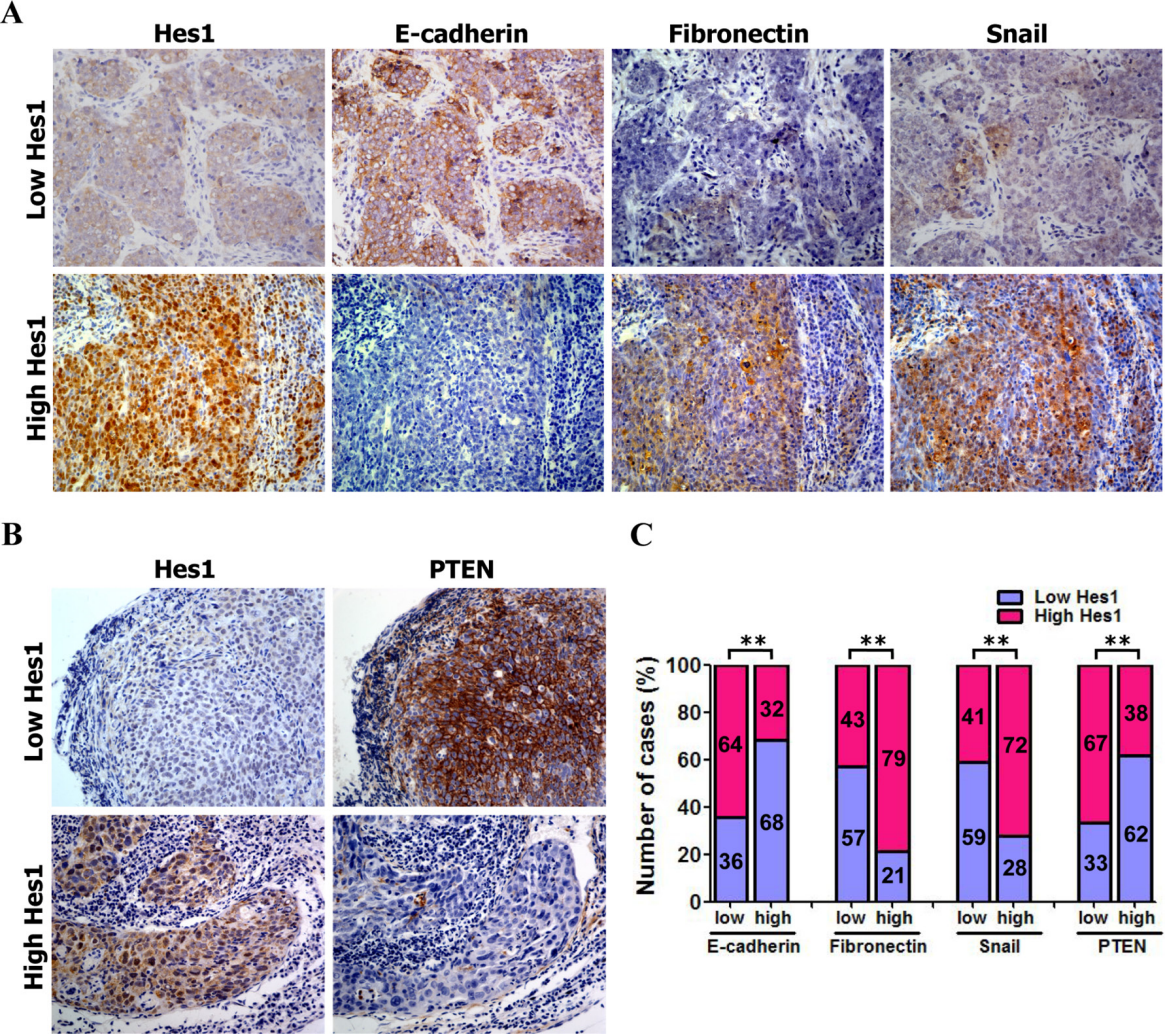
Association between the expression of Hes1 and PTEN and EMT markers **A.** Representative images of IHC staining of Hes1, E-cadherin, fibronectin, and snail in NPC biopsies. Original magnification: 400×. **B.** Association of low and high expression of Hes1 and PTEN based on IHC analysis **C.** The Hes1 expression was negatively associated with PTEN and E-cadherin expression, and positively associated with fibronectin and snail expression.

As mentioned above, Hes1 expression was increased in NPC biopsies (Figure 1A, 1B; Supplementary Table S1). Our study (Supplementary Table 3) and previous studies [47, 48] observed reduced expression of PTEN in NPC tissues [47–49]. Therefore, we used IHC to identify any association between the expression of PETN and Hes1 in NPC biopsies. The expression level of Hes1 and PTEN was analyzed in 103 cases of human primary NPC tissues based on IHC. We found that a negative association between PTEN and Hes1 expression in the 103 NPC biopsies (Figure 6B, 6C and Supplementary Table S4).

**Figure 7:**
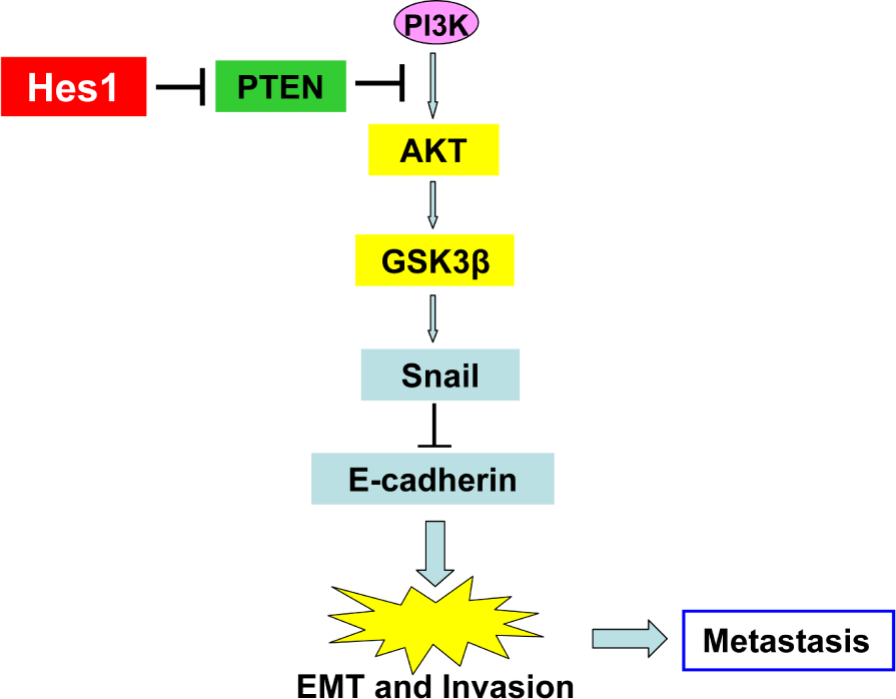
A proposed model of the Hes1/PTEN axis in the regulation of EMT, invasion, and metastasis of NPC cells

Furthermore, high expression of Hes1 is associated with high expression of fibronectin and snail, and low expression of E-cadherin in the NPC biopsies (Figure 6A, 6C and Supplementary Table S4). Statistical analyses revealed a significant negative association between E-cadherin and Hes1 expression, and a significant positive association between both fibronectin and snail and Hes1 expression in NPC biopsies (Figure 6A, 6C and Supplementary Table S4). These results suggest that high expression of fibronectin and snail and low expression of PTEN and E-cadherin were significantly associated with Hes1 overexpression in NPC cells undergoing EMT, invasion, and metastasis. Taken together, our results revealed a negative association between high expression of Hes1/fibronectin/snail and low expression of PTEN/E-cadherin in NPC cells, supporting a model in which Hes1 transcriptionally inhibits PTEN expression to activate the PI3K/Akt/GSK-3β/snail signaling pathway and downregulate E-cadherin (Figure 7).

## DISCUSSION

Although double immunofluorescence have shown that Hes1, a downstream target of Notch1 signaling, was expressed in Sox2- and Oct4-positive cells in human primary NPC tissue [50], the present study was the first one demonstrating that the expression of Hes1 was significantly increased in NPC biopsies compared to normal tissues. Increased levels of Hes1 mRNA and/or protein have also been observed in a number of solid cancers [20–30]. To our knowledge, the present study was also the first one reporting that Hes1 expression is associated with the invasion and metastasis of human NPC, such as the tumor size (T classification), lymph node metastasis (N classification), and distant metastasis (M classification), which has never been reported in NPC and other cancers. Previous studies have reported that Hes1 overexpression was associated with poor survival of patients with glioma [25] and medulloblastomas [30]. Results of these studies and the present study suggest that Hes1 overexpression is a common feature in human cancers and the expression of Hes1 may be served as a helpful prognostic marker to evaluate the prognosis of NPC patients.

In the present study, we observed Hes1 expression in both cytoplasm and nuclei of malignant and nonmalignant cells and tissues, which is consistent with the previous reports in colorectal cancer [20], pancreatic cancer [31], and rhabdomyosarcoma [51]. These results suggest that Hes1 was synthesized in cytoplasm and transported into nuclei to function as a transcriptional repressor. However, the dynamic distribution and biological activities of Hes1 in the development of NPC need to be further investigated.

Previous study reported that the expression of Hes1 in C4–2B cells, a highly metastatic derivative of the LNCaP cell line, was 20-fold higher than that in LNCaP cells [34]. In our study, we identified the association of high expression of Hes1 and the invasion and metastasis in 103 NPC cases. Gain-of-function and loss-of-function studies demonstrated that high expression of Hes1 was associated with improved migration and invasion of NPC cells *in vitro*. In addition, exogenous expression of Hes1 led to invasion and metastasis of NPC cells in mouse NPC model. These results strongly support the role of Hes1 in invasion and metastasis of NPC. The previous studies reported an association between Bmi-1 expression and local invasion of primary tumor of NPC. However, no association between Bmi-1 expression and metastasis was identified in NPC biopsies, suggesting that overexpression of Bmi-1 alone is not sufficient to induce metastasis of NPC, and Bmi-1 is mainly involved in local invasion of tumors [45, 52]. In addition, it has been shown that Bmi-1 overexpression trigged EMT to improve the invasion property of NPC cells [45, 52]. In the present study, our results suggest that overexpression of Hes1 alone is sufficient to induce local invasion and metastasis of NPC. Therefore, Hes1 may play a more important role in the invasion and metastasis of NPC than Bmi-1.

The majority of cancer deaths are due to tumor metastasis, therefore, metastasis prevention of tumor cells has received much attention [35–39]. Understanding the molecular mechanisms underlying cancer invasion and metastasis may lead to novel therapeutic approaches against cancers [35–39]. EMT is an early and characteristic event in the invasion and metastasis of various cancers [36, 40]. In the present study, we found that upregulation of Hes1 triggered EMT-like cellular marker alterations and enhanced the motility and invasion of NPC cells, whereas silencing endogenous Hes1 expression reversed EMT-like phenotypes and reduced cell motility and invasion. Therefore, Hes1 may be an important target for the prevention of invasion and metastasis of NPC.

In our previous studies, expression profiling of various biomarkers involved in EMT was performed using tissue microarray-based immunohistochemistry in 122 clinical specimens of NPC [53, 54]. Our results showed that neoplastic spindle cells in the clinical specimens of NPC exhibited EMT features [54]. In addition, we found that up-regulation of EMT markers (vimentin, fibronectin, MMP-2, periostin, SPARC, snail, and slug), and E- to N-cadherin switch, occurred preferentially in tumors containing a large proportion of spindle-shaped malignant cells [53, 54]. Therefore, in the present study, we investigated the expression of Hes1 and EMT biomarkers in a larger cohort of 103 NPC samples from the previous studies [53, 54] to understand the clinical correlation between Hes1 expression and EMT biomarkers. Our results demonstrated that the high expression of fibronectin and snail and the low expression of E-cadherin were significantly associated with the gain of Hes1 expression in EMT, invasion and metastasis of NPC, suggesting that Hes1 plays an important role in triggering EMT in NPC specimens, which was consistent with the results of *in vitro* studies described above. To the best of our knowledge, this is the first study identifying the essential role of Hes1 in induction of EMT-like phenotypes, invasion and metastasis of cancers.

It is well known that cells undergoing EMT change from a cobblestone-like appearance to an elongated and mesenchymal phenotype [35–39]. The previous report showed that although no EMT-associated morphological changes were observed in Bmi-1-overexpressing immortalized human mammary epithelial cells (HMECs), Bmi-1 overexpression in immortalized HMECs repressed epithelial markers and up-regulated mesenchymal markers, and promoted cellular motility and invasion [55]. Conversely, the repression of Bmi-1 reversed the expression of EMT markers [55]. It is very clearly that Bmi-1 alone is not sufficient to induce the typical EMT morphological changes in immortalized HMECs. Furthermore, the morphologic changes of EMT might be directed by differential oncogene activation. Ras [56] and ILEI [57] can lead to EMT, tumor formation and metastasis. These findings suggest that additional oncogenic events, such as H-Ras expression or loss of expression of tumor suppressor genes could be involved in the EMT of immortalized HMECs induced by Bmi-1. Thus, these authors [55] thought that Bmi-1-induced EMT is cell-type specific. In this study, Hes1 overexpression didn't induce the typical EMT morphological changes in NPC cells, but increased the motility and invasive properties of NPC cells, which is concurrent with the increased expression of mesenchymal markers and the decreased expression of epithelial markers. More importantly, high expression of Hes1 is associated with high expression of mesenchymal markers (fibronectin and snail), and low expression of E-cadherin in the NPC biopsies. Together, Hes1 alone is not sufficient to trigger the typical EMT morphological changes (i.e., mesenchymal-like morphological conversion) in NPC cell types (CNE2 and SUNE1 cells) examined.

β-catenin is frequently used as a mesenchymal marker gene as a target of WNT pathway. In our study, Western blot analysis and qRT-PCR illustrated that ectopic expression of Hes1 in NPC cells significantly reduced β-catenin expression (Figure 2). Our observations revealed that Hes1 overexpression causes the reduced expression of membranous and cytoplasmic β-catenin, and didn't lead to nuclear β-catenin accumulation in tumor section formed from Hes1-expressing CNE2 cells (Supplementary Figure S3). In NPC, β-catenin is used as both mesenchymal marker gene [58–61] and epithelial marker gene [62, 63]. Thus, in this case, we suggest that β-catenin can be used as an epithelial marker involved in Hes1-induced EMT-like cellular marker alterations.

Subsequently, we identified PTEN as a potential target of Hes1. It has been reported that loss of function or downregulation of PTEN was associated with metastasis, more aggressive growth behaviors, and/or poor prognostic phenotypes of many cancers including NPC [45, 48, 64–73]. In the present study, we provided evidence showing that PTEN was a potentially functional target of Hes1 and involved in the metastasis and invasion of NPC. First, a negative correlation between Hes1 and PTEN expression was observed in NPC cells and specimens. Second, suppression of PTEN expression by shRNA in Hes1-repressed CNE2 cells rescued PI3K/AKT activities and the migration/invasion of cancer cells. In addition, we found that Hes1 bound to the promoter region of PTEN. Moreover, the promoter luciferase reporter assays revealed that the DNA sequence has promoter activity and Hes-1 directly regulates the promoter activity of PTEN [46]. Collectively, these results suggest that PTEN is a bona fide target of Hes1, which negatively modulates PTEN expression.

Next, we investigated whether Hes1 triggered EMT, migration and invasion of NPC cells *in vitro* through downregulating PTEN. It has been reported that PTEN was involved in the regulation of EMT during embryonic development and cancer progress by downregulating the PI3K/Akt pathway [45]. Increasing evidence supports that inactivation or downregulation of the tumor suppressor PTEN triggers EMT of cancer cells [45, 74–76], which then promotes the invasion and metastasis of various cancers including NPC [45, 75]. In the present study, we found that PTEN overexpression mimicked shHes1-induced MET, and inhibited the migration and invasion of NPC cells, whereas shRNA-PTEN expression in shHes1-expressing CNE2 cells reversed shHes1-induced MET and abrogated shHes1-mediated suppression of motility and invasion of NPC cells. In addition, it has been shown that Notch1 regulated leukemic cell growth through multiple overlapping mechanisms including downregulation of PTEN by Hes1 [77]. Furthermore, the Notch signaling indirectly regulates the PI3K pathway through a Hes1-PTEN axis during normal development of T cells [46]. These studies suggest that Hes1 is involved in different physiological and pathological processes by targeting PTEN. Taken together, Hes1 triggered EMT-like phenotypes and promoted the migration and invasion of NPC cells by downregulating PTEN, a downstream target of Hes1. However, additional Hes1 targets may also be involved in EMT, and invasion and metastasis of NPC cells.

In conclusion, we demonstrated, for the first time, that Hes1 played an important role in the pathogenesis of NPC by inducing EMT-like cellular marker alterations to promote the invasion and metastasis of NPC cells. We also provided evidence showing that Hes1 downregulated PTEN to activate the PI3K/Akt pathway, which may be one of the major mechanisms of Hes1-induced EMT-like phenotypes of NPC cells. Therefore, Hes1 may be a promising therapeutic target for the treatment of advanced NPC by inhibiting EMT, invasion and metastasis of cancer cells.

## MATERIALS AND METHODS

### Patients and tissue samples

A total of 103 paraffin-embedded NPC biopsies and 29 non-cancerous nasopharyngeal epithelial biopsies were collected in the Department of Pathology, the People's Hospital of Gaozhou City, the Affiliated Hospital of Guangdong Medical College, China, between 2003 and 2005. None of the 103 NPC patients received preoperative radiotherapy or chemotherapy. Informed consent was approved by the local Institutional Research Ethics Committee. The clinicopathologic variables and related information of NPC biopsies were collected as previously described [53, 54, 78].

### Immunohistochemistry (IHC)

After deparaffinization and rehydration, the paraffin-embedded sections were subjected to high pressure for 2 min for antigenic retrieval. The slides were incubated overnight at 4°C with the following primary antibodies: Hes1 (Bioss, dilution 1:500), PTEN (Cell Signaling, dilution 1:200), E-cadherin (BD, dilution 1:300), Fibronectin (Abcam, dilution 1:300), and Snail (Cell Signaling, clone C15D3, dilution 1:100). PBS were used as negative controls. The sections were then incubated with DAB for 2 min. The staining intensity of tumor cells was grouped into four grades: 0, no staining; 1, weak staining; 2, modest staining; and 3, strong staining. The positive staining ratio of tumor cells was classified into four grades: 0, no positive tumor cells; 1, < 10% positive tumor cells; 2, 10–50% positive tumor cells; and 3, > 50% positive tumor cells. The positive staining ratio of tumor cells = hes1-positive tumor cells/total tumor cellsX100%. The general IHC results were calculated by multiplying the positive staining grade by the intensity grade (0, 1, 2, 3, 4, 6, and 9). Finally, general IHC results ≦4 and ≧6 were defined as low and high expression, respectively. Two pathologists examined and scored IHC results blindly without knowing the clinical characteristics and prognosis.

### Cell lines and cell culture

Human NPC cell lines CNE1, CNE2, HNE1, SUNE1, 5–10F, 6–10B, and NP69 cell lines were kindly provided from Prof. Qiao Tao (Chinese University of Hong Kong, Hong Kong, China), Prof. Yixin Zeng (Sun Yat-sen University, Guangzhou, China), and Prof. Musheng Zeng (Sun Yat-sen University, Guangzhou, China). CNE1, CNE2, HNE1, SUNE1, 5–10F, 6–10B cells were cultured in RPMI 1640 medium supplemented with 10% fetal bovine surum (FBS) in a humidified incubator with 5% CO_2_ at 37°C, while NP69 cell line was maintained in keratinocyte/serum-free medium (Invitrogen).

### Plasmids, lentivirus production, and transduction

The plasmid pWPXL-Hes1 was obtained from Addgene (Addgene plasmid 36983). The pCSII vectors, carrying shRNA for Hes1 knockdown or scrambled sequence, were generously provided by Prof. Ryoichiro Kageyama (Kyoto University, Kyoto, Japan) [8]. pFUCW-shPTEN [79] was a gift from Prof. Bryan W. Luikart (Oregon Health and Science University, Portland, USA). The plasmid pLenti-GIII-CMV-hPTEN-RFP-2A-Puro was purchased from Applied Biological Materials (ABM) Inc (Canada). The lentiviral packaging plasmids psPAX2 and pMD2.G were kindly provided by Prof. Didier Trono (University of Geneva, Geneva, Switzerland). To generate stable cell lines, recombinant lentiviruses (namely LV-con, LV-Hes1, LV-shSCR, LV-shHes1, LV-shPTEN, and LV-PTEN) were generated as previously described [80], and subsequently used to infect CNE2 and SUNE1 cells.

### RNA isolation and quantitative real-time PCR (qRT-PCR)

Total RNA was extracted from NPC cells using Trizol Reagent (TaKaRa, Dalian, China) according to the manufacturer's instruction. Then mRNA was reversely transcribed to cDNA using the PrimeScript RT reagent Kit (TaKaRa). To evaluate the mRNA levels of a number of genes, qRT-PCR was performed on a Stratagene Mx3005P qRT-PCR System using SYBR Green qRT-PCR master mix (TaKaRa). GAPDH was used as the internal control. The primers used in qRT-PCR assay were listed in Supplementary Table S5. All samples were normalized to internal controls and fold changes were calculated based on relative quantification (2^−ΔΔCt^).

### Western blot analysis

Protein lysates were separated by sodium dodecyl sulfate polyacrylamide gel electrophoresis (SDS-PAGE), and transferred to a polyvinylidene difluoride (PVDF) membrane. The blots were probed with the primary antibodies against β-actin, Hes1 (Bioss), E-cadherin, α-catenin, N-cadherin, vimentin, fibronectin (BD Biosciences), β-catenin, snail, PTEN, AKT, p-AKT, GSK3β, or p-GSK3β (Cell Signaling Technology), followed by HRP (horseradish peroxidase)-labeled secondary antibodies. The hybridization signal was detected using enhanced chemiluminescence (ECL). β-actin was used as a loading control.

### Transwell migration and boyden invasion assays

For transwell migration assay, 1 × 10^5^ cells were seeded into the upper chamber (with 8.0 μm pores, BD Biosciences) in serum-free RPMI 1640. Boyden invasion assay was performed using matrigel (BD Biosciences) in the upper chamber. RPMI 1640 with 10% FBS was loaded in the lower compartment as chemo-attractant. After 20 hours, the migrated or invaded cells were fixed with 100% methanol, stained with hematoxylin solution (Sigma), and counted in five randomly selected optical fields.

### *In vivo* metastasis analysis in nude mice

Female BALB/c nude mice (4–5 weeks) were purchased from the Medical Laboratory Animal Center of Guangdong Province, and maintained in microisolator cages under aseptic conditions. For *in vivo* metastasis assays, the vector- or Hes1-expressing CNE2 cells (1.0 × 10^6^) were subcapsularly transplanted into the liver of nude mice (8 mice/per group), respectively. All animals were sacrificed on the thirtieth day after transplantation and liver and lung were collected for metastasis analysis. This study was carried out in accordance with the Guide for the Care and Use of Laboratory Animals of the Southern Medical University. The protocol was approved by the Committee on the Ethics of Animal Experiments of the Southern Medical University. Subcapsular transplantion of NPC cells was performed under sodium pentobarbital anesthesia to minimize suffering.

### Chromatin immunoprecipitation (ChIP)

ChIP assay was performed to identify Hes1 binding sites on PTEN promoter in CNE2 cells using the Pierce Agarose Thermo ChIP Kit (Thermo) according to the manufacturer's instruction. Briefly, cross-linking was performed by adding formaldehyde (final concentration 1%) and incubated at room temperature for 10 minutes. Cross-linking reaction was terminated by the addition of glycine solution. Cells were washed with ice-cold PBS containing 0.1 mM PMSF. Cell pellets were collected by centrifugation at 3000 g for 5 minute and resuspended in 1 ml of ChIP sonication buffer. DNA was sheared by sonication and the cell debris was pelleted by centrifugation at 9,000 g for 3 minutes. Equal aliquots of chromatin supernatants were subjected to overnight immunoprecipitation with anti-Hes1 antibody (Abcam) or IgG antibody (negative control). The primer sets used in quantitative real-time PCR were listed in Supplementary Table S6.

### Statistical analyses

Statistical analyses were performed using the SPSS 13.0 software package. The χ^2^ test was used to analyze the association between clinicopathological characteristics and Hes1 expression. Two-tailed Student's *t* test was used for comparisons of two independent groups. The data were presented as mean ± SEM. The “*” sign denotes *P* < 0.05 compared with control and the “**” sign denotes *P* < 0.01 compared with control.

## SUPPLEMENTARY FIGURES AND TABLES



## References

[1] Tan SL, Ohtsuka T, Gonzalez A, Kageyama R (2012). MicroRNA9 regulates neural stem cell differentiation by controlling Hes1 expression dynamics in the developing brain. Genes Cells.

[2] Bai G, Sheng N, Xie Z, Bian W, Yokota Y, Benezra R, Kageyama R, Guillemot F, Jing N (2007). Id sustains Hes1 expression to inhibit precocious neurogenesis by releasing negative autoregulation of Hes1. Dev Cell.

[3] Hirata H, Tomita K, Bessho Y, Kageyama R (2001). Hes1 and Hes3 regulate maintenance of the isthmic organizer and development of the mid/hindbrain. Embo J.

[4] Nakamura Y, Sakakibara S, Miyata T, Ogawa M, Shimazaki T, Weiss S, Kageyama R, Okano H (2000). The bHLH gene hes1 as a repressor of the neuronal commitment of CNS stem cells. J Neurosci.

[5] Piper M, Barry G, Hawkins J, Mason S, Lindwall C, Little E, Sarkar A, Smith AG, Moldrich RX, Boyle GM, Tole S, Gronostajski RM, Bailey TL (2010). NFIA controls telencephalic progenitor cell differentiation through repression of the Notch effector Hes1. J Neurosci.

[6] Sato T, Shimazaki T, Naka H, Fukami S, Satoh Y, Okano H, Lax I, Schlessinger J, Gotoh N (2010). FRS2alpha regulates Erk levels to control a self-renewal target Hes1 and proliferation of FGF-responsive neural stem/progenitor cells. Stem Cells.

[7] Kayahara T, Sawada M, Takaishi S, Fukui H, Seno H, Fukuzawa H, Suzuki K, Hiai H, Kageyama R, Okano H, Chiba T (2003). Candidate markers for stem and early progenitor cells, Musashi-1 and Hes1, are expressed in crypt base columnar cells of mouse small intestine. FEBS Lett.

[8] Kobayashi T, Kageyama R (2010). Hes1 regulates embryonic stem cell differentiation by suppressing Notch signaling. Genes Cells.

[9] Kobayashi T, Mizuno H, Imayoshi I, Furusawa C, Shirahige K, Kageyama R (2009). The cyclic gene Hes1 contributes to diverse differentiation responses of embryonic stem cells. Genes Dev.

[10] Kunisato A, Chiba S, Nakagami-Yamaguchi E, Kumano K, Saito T, Masuda S, Yamaguchi T, Osawa M, Kageyama R, Nakauchi H, Nishikawa M, Hirai H (2003). HES-1 preserves purified hematopoietic stem cells *ex vivo* and accumulates side population cells *in vivo*. Blood.

[11] Moriyama M, Osawa M, Mak SS, Ohtsuka T, Yamamoto N, Han H, Delmas V, Kageyama R, Beermann F, Larue L, Nishikawa S (2006). Notch signaling via Hes1 transcription factor maintains survival of melanoblasts and melanocyte stem cells. J Cell Biol.

[12] Riccio O, van Gijn ME, Bezdek AC, Pellegrinet L, van Es JH, Zimber-Strobl U, Strobl LJ, Honjo T, Clevers H, Radtke F (2008). Loss of intestinal crypt progenitor cells owing to inactivation of both Notch1 and Notch2 is accompanied by derepression of CDK inhibitors p27Kip1 and p57Kip2. EMBO Rep.

[13] Stanger BZ, Stiles B, Lauwers GY, Bardeesy N, Mendoza M, Wang Y, Greenwood A, Cheng KH, McLaughlin M, Brown D, Depinho RA, Wu H, Melton DA (2005). Pten constrains centroacinar cell expansion and malignant transformation in the pancreas. Cancer Cell.

[14] Manosalva I, Gonzalez A, Kageyama R (2013). Hes1 in the somatic cells of the murine ovary is necessary for oocyte survival and maturation. Dev Biol.

[15] Kageyama R, Ohtsuka T, Tomita K (2000). The bHLH gene Hes1 regulates differentiation of multiple cell types. Mol Cells.

[16] Sang L, Coller HA (2009). Fear of commitment: Hes1 protects quiescent fibroblasts from irreversible cellular fates. Cell Cycle.

[17] Sang L, Coller HA, Roberts JM (2008). Control of the reversibility of cellular quiescence by the transcriptional repressor HES1. Science.

[18] Sang L, Roberts JM, Coller HA (2010). Hijacking HES1: how tumors co-opt the anti-differentiation strategies of quiescent cells. Trends Mol Med.

[19] Ueo T, Imayoshi I, Kobayashi T, Ohtsuka T, Seno H, Nakase H, Chiba T, Kageyama R (2012). The role of Hes genes in intestinal development, homeostasis and tumor formation. Development.

[20] Candy PA, Phillips MR, Redfern AD, Colley SM, Davidson JA, Stuart LM, Wood BA, Zeps N, Leedman PJ (2013). Notch-induced transcription factors are predictive of survival and 5-fluorouracil response in colorectal cancer patients. Br J Cancer.

[21] Gao F, Zhang Y, Wang S, Liu Y, Zheng L, Yang J, Huang W, Ye Y, Luo W, Xiao D (2014). Hes1 is involved in the self-renewal and tumourigenicity of stem-like cancer cells in colon cancer. Sci Rep.

[22] Peignon G, Durand A, Cacheux W, Ayrault O, Terris B, Laurent-Puig P, Shroyer NF, Van Seuningen I, Honjo T, Perret C, Romagnolo B (2011). Complex interplay between beta-catenin signalling and Notch effectors in intestinal tumorigenesis. Gut.

[23] Farnie G, Clarke RB, Spence K, Pinnock N, Brennan K, Anderson NG, Bundred NJ (2007). Novel cell culture technique for primary ductal carcinoma *in situ*: role of Notch and epidermal growth factor receptor signaling pathways. J Natl Cancer Inst.

[24] Giovannini C, Gramantieri L, Minguzzi M, Fornari F, Chieco P, Grazi GL, Bolondi L (2012). CDKN1C/P57 is regulated by the Notch target gene Hes1 and induces senescence in human hepatocellular carcinoma. Am J Pathol.

[25] Chen L, Zhang W, Yan W, Han L, Zhang K, Shi Z, Zhang J, Wang Y, Li Y, Yu S, Pu P, Jiang C, Jiang T (2012). The putative tumor suppressor miR-524-5p directly targets Jagged-1 and Hes-1 in glioma. Carcinogenesis.

[26] Konishi J, Kawaguchi KS, Vo H, Haruki N, Gonzalez A, Carbone DP, Dang TP (2007). Gamma-secretase inhibitor prevents Notch3 activation and reduces proliferation in human lung cancers. Cancer Res.

[27] Sun W, Gaykalova DA, Ochs MF, Mambo E, Arnaoutakis D, Liu Y, Loyo M, Agrawal N, Howard J, Li R, Ahn S, Fertig E, Sidransky D (2014). Activation of the NOTCH pathway in head and neck cancer. Cancer Res.

[28] Hopfer O, Zwahlen D, Fey MF, Aebi S (2005). The Notch pathway in ovarian carcinomas and adenomas. Br J Cancer.

[29] Cuevas IC, Slocum AL, Jun P, Costello JF, Bollen AW, Riggins GJ, McDermott MW, Lal A (2005). Meningioma transcript profiles reveal deregulated Notch signaling pathway. Cancer Res.

[30] Fiaschetti G, Abela L, Nonoguchi N, Dubuc AM, Remke M, Boro A, Grunder E, Siler U, Ohgaki H, Taylor MD, Baumgartner M, Shalaby T, Grotzer MA (2014). Epigenetic silencing of miRNA-9 is associated with HES1 oncogenic activity and poor prognosis of medulloblastoma. Br J Cancer.

[31] Hingorani SR, Petricoin EF, Maitra A, Rajapakse V, King C, Jacobetz MA, Ross S, Conrads TP, Veenstra TD, Hitt BA, Kawaguchi Y, Johann D, Liotta LA (2003). Preinvasive and invasive ductal pancreatic cancer and its early detection in the mouse. Cancer Cell.

[32] Bolos V, Mira E, Martinez-Poveda B, Luxan G, Canamero M, Martinez-A C, Manes S, de la Pompa JL (2013). Notch activation stimulates migration of breast cancer cells and promotes tumor growth. Breast Cancer Res.

[33] Larson GA, Chen Q, Kugel DS, Ge Y, LaFiura K, Haska CL, Cherian C, Devidas M, Linda SB, Taub JW, Matherly LH (2009). The impact of NOTCH1, FBW7 and PTEN mutations on prognosis and downstream signaling in pediatric T-cell acute lymphoblastic leukemia: a report from the Children's Oncology Group. Leukemia.

[34] Scorey N, Fraser SP, Patel P, Pridgeon C, Dallman MJ, Djamgoz MB (2006). Notch signalling and voltage-gated Na+ channel activity in human prostate cancer cells: independent modulation of *in vitro* motility. Prostate Cancer Prostatic Dis.

[35] Kalluri R, Weinberg RA (2009). The basics of epithelial-mesenchymal transition. J Clin Invest.

[36] Thiery JP, Sleeman JP (2006). Complex networks orchestrate epithelial-mesenchymal transitions. Nat Rev Mol Cell Biol.

[37] Yilmaz M, Christofori G (2009). EMT, the cytoskeleton, and cancer cell invasion. Cancer Metastasis Rev.

[38] Li J, Yang S, Yan W, Yang J, Qin YJ, Lin XL, Xie RY, Wang SC, Jin W, Gao F, Shi JW, Zhao WT, Jia JS (2015). MicroRNA-19 triggers epithelial-mesenchymal transition of lung cancer cells accompanied by growth inhibition. Lab Invest.

[39] Wang SC, Lin XL, Li J, Zhang TT, Wang HY, Shi JW, Yang S, Zhao WT, Xie RY, Wei F, Qin YJ, Chen L, Yang J (2014). MicroRNA-122 triggers mesenchymal- epithelial transition and suppresses hepatocellular carcinoma cell motility and invasion by targeting RhoA. PLoS One.

[40] Christofori G (2006). New signals from the invasive front. Nature.

[41] De Obaldia ME, Bell JJ, Wang X, Harly C, Yashiro-Ohtani Y, DeLong JH, Zlotoff DA, Sultana DA, Pear WS, Bhandoola A (2013). T cell development requires constraint of the myeloid regulator C/EBP-alpha by the Notch target and transcriptional repressor Hes1. Nat Immunol.

[42] Yan B, Raben N, Plotz PH (2002). Hes-1, a known transcriptional repressor, acts as a transcriptional activator for the human acid alpha-glucosidase gene in human fibroblast cells. Biochem Biophys Res Commun.

[43] Blanco-Aparicio C, Renner O, Leal JF, Carnero A (2007). PTEN, more than the AKT pathway. Carcinogenesis.

[44] Hill R, Wu H (2009). PTEN, stem cells, and cancer stem cells. J Biol Chem.

[45] Song LB, Li J, Liao WT, Feng Y, Yu CP, Hu LJ, Kong QL, Xu LH, Zhang X, Liu WL, Li MZ, Zhang L, Kang TB (2009). The polycomb group protein Bmi-1 represses the tumor suppressor PTEN and induces epithelial-mesenchymal transition in human nasopharyngeal epithelial cells. J Clin Invest.

[46] Wong GW, Knowles GC, Mak TW, Ferrando AA, Zuniga-Pflucker JC (2012). HES1 opposes a PTEN-dependent check on survival, differentiation, and proliferation of TCRbeta-selected mouse thymocytes. Blood.

[47] Li J, Gong P, Lyu X, Yao K, Li X, Peng H (2014). Aberrant CpG island methylation of PTEN is an early event in nasopharyngeal carcinoma and a potential diagnostic biomarker. Oncol Rep.

[48] Xu X, Yang H, Huo X (2004). Expression and significance of PTEN in nasopharyngeal carcinoma]. Lin Chuang Er Bi Yan Hou Ke Za Zhi.

[49] Zhang LY, Ho-Fun LV, Wong AM, Kwong DL, Zhu YH, Dong SS, Kong KL, Chen J, Tsao SW, Guan XY, Fu L (2013). MicroRNA-144 promotes cell proliferation, migration and invasion in nasopharyngeal carcinoma through repression of PTEN. Carcinogenesis.

[50] Zhang Y, Peng J, Zhang H, Zhu Y, Wan L, Chen J, Chen X, Lin R, Li H, Mao X, Jin K (2010). Notch1 signaling is activated in cells expressing embryonic stem cell proteins in human primary nasopharyngeal carcinoma. J Otolaryngol Head Neck Surg.

[51] Roma J, Masia A, Reventos J, Sanchez DTJ, Gallego S (2011). Notch pathway inhibition significantly reduces rhabdomyosarcoma invasiveness and mobility *in vitro*. Clin Cancer Res.

[52] Song LB, Zeng MS, Liao WT, Zhang L, Mo HY, Liu WL, Shao JY, Wu QL, Li MZ, Xia YF, Fu LW, Huang WL, Dimri GP (2006). Bmi-1 is a novel molecular marker of nasopharyngeal carcinoma progression and immortalizes primary human nasopharyngeal epithelial cells. Cancer Res.

[53] Luo W, Fang W, Li S, Yao K (2012). Aberrant expression of nuclear vimentin and related epithelial-mesenchymal transition markers in nasopharyngeal carcinoma. Int J Cancer.

[54] Luo W, Li S, Peng B, Ye Y, Deng X, Yao K (2013). Embryonic stem cells markers SOX2, OCT4 and Nanog expression and their correlations with epithelial-mesenchymal transition in nasopharyngeal carcinoma. PLoS One.

[55] Guo BH, Feng Y, Zhang R, Xu LH, Li MZ, Kung HF, Song LB, Zeng MS (2011). Bmi-1 promotes invasion and metastasis, and its elevated expression is correlated with an advanced stage of breast cancer. Mol Cancer.

[56] Grunert S, Jechlinger M, Beug H (2003). Diverse cellular and molecular mechanisms contribute to epithelial plasticity and metastasis. Nat Rev Mol Cell Biol.

[57] Waerner T, Alacakaptan M, Tamir I, Oberauer R, Gal A, Brabletz T, Schreiber M, Jechlinger M, Beug H (2006). ILEI: a cytokine essential for EMT, tumor formation, and late events in metastasis in epithelial cells. Cancer Cell.

[58] Cai LM, Lyu XM, Luo WR, Cui XF, Ye YF, Yuan CC, Peng QX, Wu DH, Liu TF, Wang E, Marincola FM, Yao KT, Fang WY (2015). EBV-miR-BART7-3p promotes the EMT and metastasis of nasopharyngeal carcinoma cells by suppressing the tumor suppressor PTEN. Oncogene.

[59] Zong D, Yin L, Zhong Q, Guo WJ, Xu JH, Jiang N, Lin ZR, Li MZ, Han P, Xu L, He X, Zeng MS (2015). ZNF 488 Enhances the Invasion and Tumorigenesis in Nasopharyngeal Carcinoma via the Wnt Signaling Pathway Involving Epithelial Mesenchymal Transition. Cancer Res Treat.

[60] Lin Z, Wan X, Jiang R, Deng L, Gao Y, Tang J, Yang Y, Zhao W, Yan X, Yao K, Sun B, Chen Y (2014). Epstein-Barr virus-encoded latent membrane protein 2A promotes the epithelial-mesenchymal transition in nasopharyngeal carcinoma via metastatic tumor antigen 1 and mechanistic target of rapamycin signaling induction. J Virol.

[61] Luo WR, Chen XY, Li SY, Wu AB, Yao KT (2012). Neoplastic spindle cells in nasopharyngeal carcinoma show features of epithelial-mesenchymal transition. Histopathology.

[62] Zhang P, Liu H, Xia F, Zhang QW, Zhang YY, Zhao Q, Chao ZH, Jiang ZW, Jiang CC (2014). Epithelial-mesenchymal transition is necessary for acquired resistance to cisplatin and increases the metastatic potential of nasopharyngeal carcinoma cells. Int J Mol Med.

[63] Lin JC, Liao SK, Lee EH, Hung MS, Sayion Y, Chen HC, Kang CC, Huang LS, Cherng JM (2009). Molecular events associated with epithelial to mesenchymal transition of nasopharyngeal carcinoma cells in the absence of Epstein-Barr virus genome. J Biomed Sci.

[64] Abate-Shen C, Banach-Petrosky WA, Sun X, Economides KD, Desai N, Gregg JP, Borowsky AD, Cardiff RD, Shen MM (2003). Nkx3.1, Pten mutant mice develop invasive prostate adenocarcinoma and lymph node metastases. Cancer Res.

[65] Akca H, Demiray A, Tokgun O, Yokota J (2011). Invasiveness and anchorage independent growth ability augmented by PTEN inactivation through the PI3K/AKT/NFkB pathway in lung cancer cells. Lung Cancer.

[66] Chung MJ, Jung SH, Lee BJ, Kang MJ, Lee DG (2004). Inactivation of the PTEN gene protein product is associated with the invasiveness and metastasis, but not angiogenesis, of breast cancer. Pathol Int.

[67] Damsky WE, Curley DP, Santhanakrishnan M, Rosenbaum LE, Platt JT, Gould RB, Taketo MM, Dankort D, Rimm DL, McMahon M, Bosenberg M (2011). beta-catenin signaling controls metastasis in Braf-activated Pten-deficient melanomas. Cancer Cell.

[68] Dankort D, Curley DP, Cartlidge RA, Nelson B, Karnezis AN, Damsky WJ, You MJ, DePinho RA, McMahon M, Bosenberg M (2009). Braf(V600E) cooperates with Pten loss to induce metastatic melanoma. Nat Genet.

[69] Ratnacaram CK, Teletin M, Jiang M, Meng X, Chambon P, Metzger D (2008). Temporally controlled ablation of PTEN in adult mouse prostate epithelium generates a model of invasive prostatic adenocarcinoma. Proc Natl Acad Sci U S a.

[70] Sawai H, Yasuda A, Ochi N, Ma J, Matsuo Y, Wakasugi T, Takahashi H, Funahashi H, Sato M, Takeyama H (2008). Loss of PTEN expression is associated with colorectal cancer liver metastasis and poor patient survival. BMC Gastroenterol.

[71] Shih MC, Chen JY, Wu YC, Jan YH, Yang BM, Lu PJ, Cheng HC, Huang MS, Yang CJ, Hsiao M, Lai JM (2012). TOPK/PBK promotes cell migration via modulation of the PI3K/PTEN/AKT pathway and is associated with poor prognosis in lung cancer. Oncogene.

[72] Sze KM, Wong KL, Chu GK, Lee JM, Yau TO, Ng IO (2011). Loss of phosphatase and tensin homolog enhances cell invasion and migration through AKT/Sp-1 transcription factor/matrix metalloproteinase 2 activation in hepatocellular carcinoma and has clinicopathologic significance. Hepatology.

[73] Zhang JG, Wang JJ, Zhao F, Liu Q, Jiang K, Yang GH (2010). MicroRNA-21 (miR-21) represses tumor suppressor PTEN and promotes growth and invasion in non-small cell lung cancer (NSCLC). Clin Chim Acta.

[74] Bowen KA, Doan HQ, Zhou BP, Wang Q, Zhou Y, Rychahou PG, Evers BM (2009). PTEN loss induces epithelial—mesenchymal transition in human colon cancer cells. Anticancer Res.

[75] Mulholland DJ, Kobayashi N, Ruscetti M, Zhi A, Tran LM, Huang J, Gleave M, Wu H (2012). Pten loss and RAS/MAPK activation cooperate to promote EMT and metastasis initiated from prostate cancer stem/progenitor cells. Cancer Res.

[76] Wang H, Quah SY, Dong JM, Manser E, Tang JP, Zeng Q (2007). PRL-3 down-regulates PTEN expression and signals through PI3K to promote epithelial-mesenchymal transition. Cancer Res.

[77] Palomero T, Sulis ML, Cortina M, Real PJ, Barnes K, Ciofani M, Caparros E, Buteau J, Brown K, Perkins SL, Bhagat G, Agarwal AM, Basso G (2007). Mutational loss of PTEN induces resistance to NOTCH1 inhibition in T-cell leukemia. Nat Med.

[78] Luo W, Yao K (2013). Molecular characterization and clinical implications of spindle cells in nasopharyngeal carcinoma: a novel molecule-morphology model of tumor progression proposed. PLoS One.

[79] Luikart BW, Schnell E, Washburn EK, Bensen AL, Tovar KR, Westbrook GL (2011). Pten knockdown *in vivo* increases excitatory drive onto dentate granule cells. J Neurosci.

[80] Shi JW, Liu W, Zhang TT, Wang SC, Lin XL, Li J, Jia JS, Sheng HF, Yao ZF, Zhao WT, Zhao ZL, Xie RY, Yang S (2013). The enforced expression of c-Myc in pig fibroblasts triggers mesenchymal-epithelial transition (MET) via F-actin reorganization and RhoA/Rock pathway inactivation. Cell Cycle.

