# Supplementary material for: Hes1 triggers epithelial-mesenchymal transition (EMT)-like cellular marker alterations and promotes invasion and metastasis of nasopharyngeal carcinoma by activating the PTEN/AKT pathway
Source: Oncotarget. 2015 Oct 2;6(34):36713–30. doi: 10.18632/oncotarget.5457 (PMC4742206; doi:10.18632/oncotarget.5457)
Supplement: Supplementary file 1 [file oncotarget-06-36713-s001.pdf]

SUPPLEMENTARY FIGURES AND TABLES

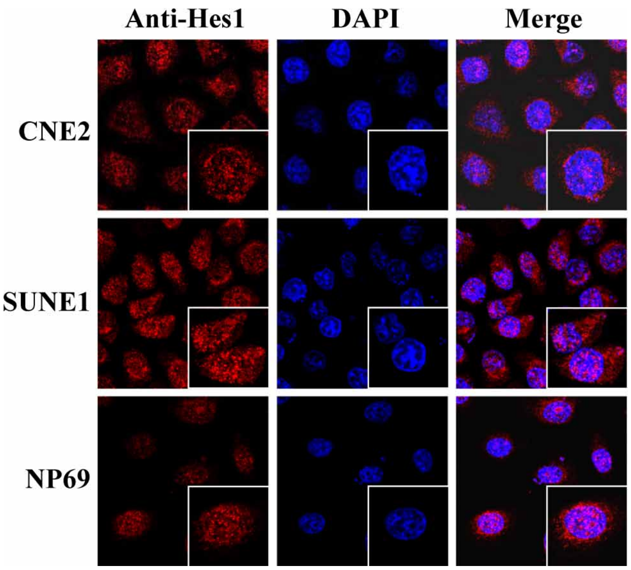

Supplementary Figure S1: Immunofluorescence assay indicated the distribution of Hes1 protein in the nucleus and cytoplasm of NP69, CNE2 and SUNE1 cells. The nuclei were stained with 4′,6-diamidino-2-phenylindole (DAPI).

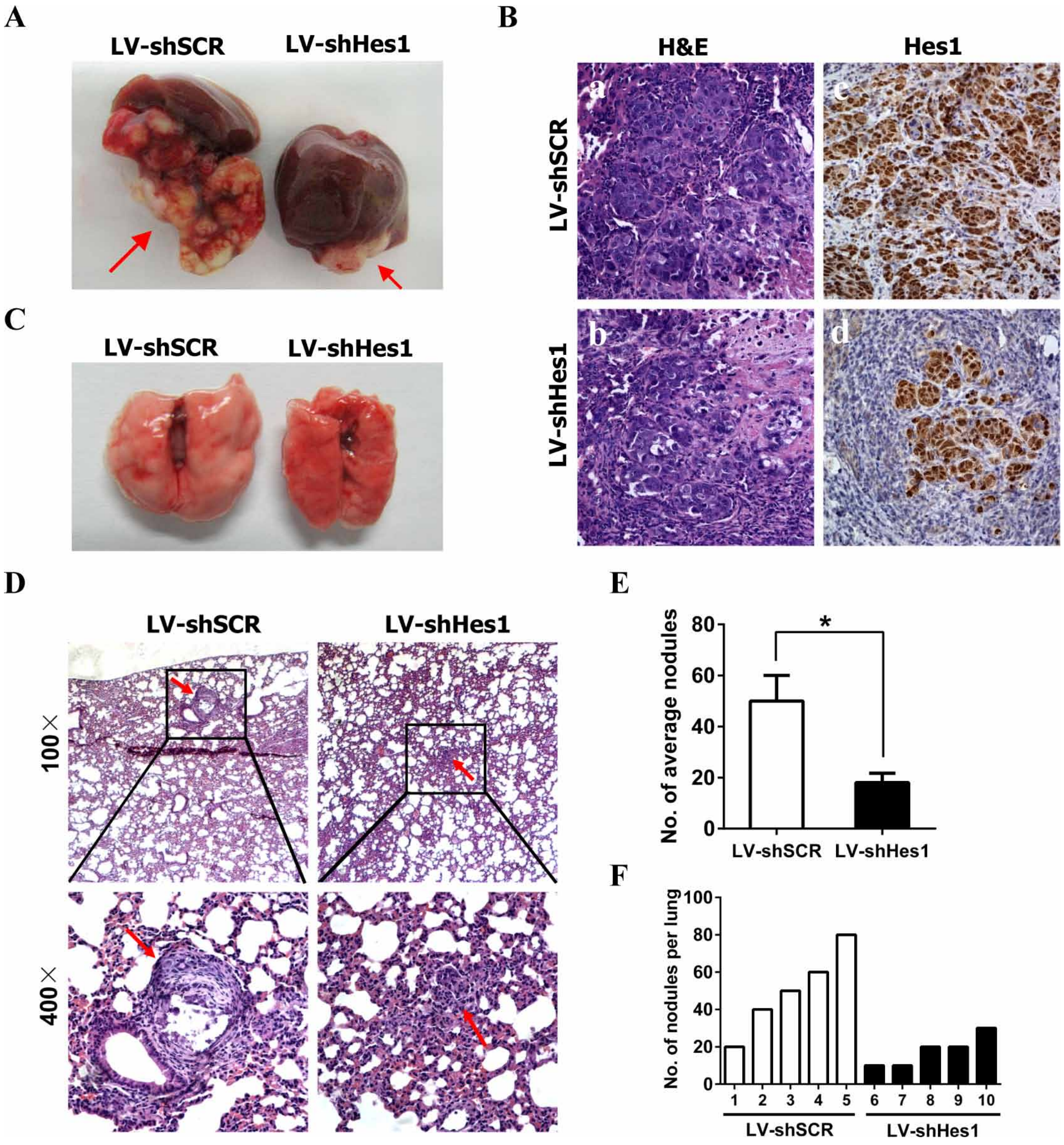

Supplementary Figure S2: RNAi-mediated silencing of endogenous Hes1 in CNE2 cells inhibited metastasis. A. Representative livers from nude mice 40 days after subcapsular liver transplantation of Hes1-shRNA- or scrambled shRNA–expressing CNE2 cells (LV-shHes1 or LV-shSCR) (5 mice/group). Arrows indicate primarily formed tumors. **B.** H&E staining (left) and IHC analysis of Hes1 expression (right) of primary tumors in the liver of nude mice (Original magnification: × 400). **C–D.** Representative lungs (C) and H&E staining of lung sections (D) of nude mice 40 days after subcapsular liver transplantation of Hes1-shRNA- or scrambled shRNA–expressing CNE2 cells. Red arrow indicates the metastatic tumor nodes. **E–F.** The number of spontaneous lung metastatic nodules in nude mice based on 10 serial sections per sample.

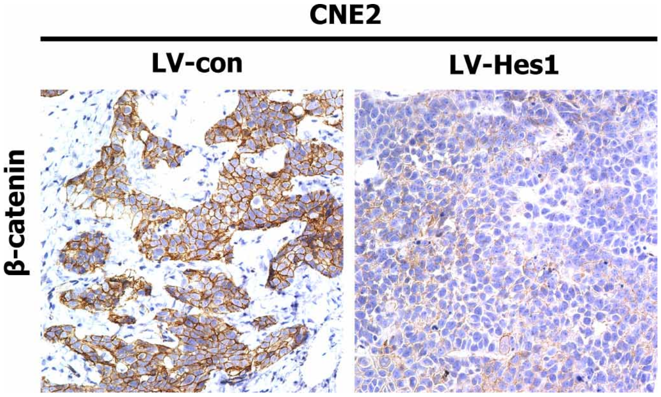

Supplementary Figure S3: The distribution of β-catenin was detected in the nucleus and cytoplasm of cancer cells by IHC. In this study, vector- or Hes1-expressing CNE2 cells were subcapsularly transplanted into the liver of nude mice and formed primary tumors in liver, followed by detecting the expression of β-catenin in formed primary tumors by immunohistochemistry (IHC).

Supplementary Table S1: Expression of Hes1 in 29 non-cancerous epithelial tissues and 103 NPC tissues

Variables	*n*	Hes1 expression		χ2	*P*
		Low (*n*, %)	High (*n*, %)		
non-cancerous epithelial tissues	29	19 (65.5)	10 (34.5)	4.715	0.03
NPC	103	44 (42.7)	59 (57.3)		

Supplementary Table S2: Univariate and multivariate analyses of the overall survival of 103 patients with NPC

Variables	Univariate analysis		Multivariate analysis	
	HR (95% CI)	*P*	HR (95% CI)	*P*
Gender				
Female vs. Male	1.135 (0.572–2.253)	0.716		
Age (y)				
< 50 vs. ≥ 50	0.917 (0.502–1.675)	0.779		
Histological type				
DNKC vs. UDC	1.010 (0.426–2.394)	0.982		
T classification				
T1-T2 vs. T3-T4	1.339 (0.730–2.456)	0.345		
N classification				
N0-N1 vs. N2-N3	2.067 (1.130–3.783)	0.019	1.120 (0.575–2.180)	0.740
M classification				
No vs. Yes	4.421 (2.209–8.847)	0.000	2.606 (1.245–5.453)	0.011
Clinical stage				
I-II vs. III-IV	3.434 (1.349–8.746)	0.010	2.035 (0.679–6.095)	0.205
Tumor recurrence				
No vs. Yes	3.619 (1.874–6.987)	0.000	4.108 (2.042–8.265)	0.000
Hes1 expression				
Low vs. High	2.803 (1.405–5.592)	0.003	2.146 (0.990–4.653)	0.053

HR: hazard ratio; 95%CI: 95% confidence interval.

Supplementary Table S3: Expression of PTEN in the 29 non-cancerous epithelial tissues and 103 NPC tissues

Variables	*n*	PTEN expression		$\chi2$	*P*
		Low (*n*, %)	High (*n*, %)		
non-cancerous epithelial tissues	29	6 (20.7)	23 (79.3)	19.772	0.001
NPC	103	69 (70.0)	34 (30.0)		

Supplementary Table S4: Association of Hes1 expression and PTEN and EMT markers in 103 NPC tissues

Variables	*n*	Hes1 expression		*P*
		Low (*n*, %)	High (*n*, %)	
E-cadherin				
Low expression	81	29 (35.8)	52 (64.2)	0.006
High expression	22	15 (68.2)	7 (31.8)	
Fibronectin				
Low expression	61	35 (57.4)	26 (42.6)	0.000
High expression	42	9 (21.4)	33 (78.6)	
Snail				
Low expression	49	29 (59.2)	20 (40.8)	0.001
High expression	54	15 (27.8)	39 (72.2)	
PTEN				
Low expression	69	23 (33.3)	46 (66.7)	0.006
High expression	34	21(61.8)	13 (38.2)	

Supplementary Table S5: Primers used in quantitative real-time PCR (qRT-PCR) assay

Gene	Forward primer (5′−3′)	Reverse primer (5′−3′)
Hes1	ACGTGCGAGGGCGTTAATAC	GGGGTAGGTCATGGCATTGA
E-cadherin	TGCCCAGAAAATGAAAAAGG	GTGTATGTGGCAATGCGTTC
β-catenin	AGGTCTGAGGAGCAGCTTCA	ATTGTCCACGCTGGATTTTC
N-cadherin	ACAGTGGCCACCTACAAAGG	CCGAGATGGGGTTGATAATG
Vimentin	GAGAACTTTGCCGTTGAAGC	GCTTCCTGTAGGTGGCAATC
PTEN	TTTGAAGACCATAACCCACCAC	ATTACACCAGTTCGTCCCTTTC
GAPDH	ACCCAGAAGACTGTGGATGG	TCTAGACGGCAGGTCAGGTC

Supplementary Table S6: Primers used in ChIP assays

ChIP-primer	Forward primer (5′−3′)	Reverse primer (5′−3′)
PTEN-A	AGGTCTCAGTCCTTTGGCTTGC	TGGTTACACAAGCACCCACATC
PTEN-B	GGGAGTGGGAATTTGGAAAG	TCAAAAGGAGGTGGAAGGAT
PTEN-C	TCCCTGCATTTCCCTCTACA	GTGCGTTGAGCAGTGTCACT
PTEN-D	GTGATGTGGCGGGACTCTTTAT	CTCTCATCTCCCTCGCCTGAG
